# Effect of lncRNA‐ATB/miR‐651‐3p/Yin Yang 1 pathway on trophoblast‐endothelial cell interaction networks

**DOI:** 10.1111/jcmm.16550

**Published:** 2021-05-04

**Authors:** Yangxue Yin, Jiashuo Zhang, Hongbiao Yu, Min Liu, Xuelian Zheng, Rong Zhou

**Affiliations:** ^1^ Department of Obstetrics and Gynecology Center for Translational Medicine Key Laboratory of Birth Defects and Related Diseases of Women and Children (Sichuan University) of Ministry of Education West China Second University Hospital Sichuan University Chengdu China; ^2^ Laboratory of Molecular Translational Medicine Center for Translational Medicine Key Laboratory of Birth Defects and Related Diseases of Women and Children (Sichuan University) Ministry of Education West China Second University Hospital Sichuan University Chengdu China

**Keywords:** human umbilical vein endothelial cells, long non‐coding RNA ATB, MiR‐651‐3p, preeclampsia, spiral artery remodelling, trophoblasts, YY1

## Abstract

Our previous studies have confirmed that lncRNA‐ATB may be involved in the pathogenesis of preeclampsia, however, it is uncertain whether lncRNA‐ATB influence the interaction between trophoblast and endothelial cells, which is crucial to the uterine spiral artery remodelling. Scratch wound healing and transwell invasion assay were conducted to test the migration and invasion of trophoblast cells. Co‐culture model was used to simulate the physiological environment in vivo. The expression levels of lncRNA‐ATB were analyzed in placenta tissues from healthy pregnant women and preeclampsia patients. Subsequently, the binding site of lncRNA‐ATB and miR‐651‐3p was verified using dual‐luciferase reporter assay, and the rescue experiment was used to study the effects of these two on the biological function. The direct effects of miR‐651‐3p and Yin Yang 1 (YY1) were verified using similar methods. LncRNA‐ATB was found to be down‐regulated in the placenta of preeclampsia patients. LncRNA‐ATB knockdown decreased trophoblast migration, invasion and colocalisation with human umbilical vein endothelial cells. MiR‐651‐3p was a direct target of lncRNA‐ATB and they had opposite effects. Moreover, the expression of lncRNA‐ATB and miR‐651‐3p in placental tissues was negatively correlated. MiR‐651‐3p has been confirmed to directly target the 3′ untranslated region of YY1. The inhibitory effects of YY1 low expression on biological function was rescued by miR‐651‐3p depletion. Western blot analysis showed that lncRNA‐ATB could regulate YY1 expression by sponging miR‐651‐3p. LncRNA‐ATB functioned as a competitive endogenous RNA of miR‐651‐3p to regulate YY1 on progress of spiral artery remodelling.

## 1 INTRODUCTION

Preeclampsia (PE) is one of the pregnancy‐specific diseases that affects 2%‐8% of all pregnancies and subsides at the end of the pregnancy.^1^ The diagnostic criteria for PE is systolic blood pressure (SBP) ≥ 140 mm Hg and/or diastolic blood pressure (DBP) ≥ 90 mm Hg, accompanied by proteinuria and/or impairment of maternal vital organ function, or fetal growth restriction.^2^ During the early stages of normal pregnancy, the extravillous trophoblasts gradually invade and replace the vascular smooth muscle cells and endothelial cells in the uterine spiral arteries, a process known as spiral artery remodelling.^3^ The pathogenesis of PE is characterized by the reduction of placental perfusion caused by shallow placental implantation, which is a consequence of disorders in spiral artery remodelling. This leads to the release of inflammatory mediators such as anti‐angiogenic factors soluble FMS‐like tyrosine kinase‐1 (sFlt‐1) and soluble endoglin (sEng), which then induce PE.^4^ The specific mechanism of PE pathogenesis remains unclear. In this study, we focus on the initial stage of PE‐improper spiral artery remodelling. We used a co‐culture model of human umbilical vein endothelial cells (HUVECs) and HTR8/SVneo cells to simulate the process of trophoblast cells replacing spiral arteries in vivo.^5^, ^6^ However, the mechanism that affect this process remains to be explored.

Epigenetics, which regulates eukaryotic gene expression, is closely associated with major human diseases, such as tumours, neurodegenerative diseases, and cardiovascular diseases.^7^, ^8^, ^9^, ^10^ Recently, there has been a lot of research interest in epigenetics in the field of life science particularly the role of non‐coding RNA in transcriptional and post‐transcriptional regulation of gene expression. Long non‐coding RNAs (lncRNAs) are a class of non‐coding RNAs that are produced by RNA polymerase II and are over 200 nucleotides in length.^11^, ^12^ Recently, several studies have shown that lncRNA play important roles in many life activities, and may be involved in the occurrence and development of diseases. He et al^13^ was the first to determine the whole‐genome lncRNAs expression pattern in PE placenta using microarray analysis. The results showed that abnormal lncRNA expression was associated with PE placenta compared to the control group. Wang et al^14^ identified 163 differentially expressed lncRNAs in placental samples of late‐onset PE patients through microarray analysis. A large number of in vitro studies have proved that these differentially expressed lncRNAs could affect the invasion, migration, tube formation, proliferation, apoptosis and other biological behaviours of trophoblast cells, such as MALAT1 and H19.^15^, ^16^ Our previous study found that lncRNA‐ATB may be a regulator of trophoblast function and is involved in the pathogenesis of PE^17^ and this study will further explore the possible mechanisms of lncRNA‐ATB action. One way in which lncRNAs participate in post‐transcriptional regulation is to recognize microRNAs (miRNAs) in the RNA‐induced silencing complex (RISC). The lncRNAs bind the miRNAs through complementary base pairing, and therefore inhibit the regulatory function of miRNAs through sponge adsorption.^18^ The mechanism of the competitive endogenous RNA in preeclampsia has been extensively studied. Liu et al^19^ found that lncRNA DLX6‐AS1 inhibited the proliferation and invasion of trophoblast cells and angiogenesis of HUVECs by targeting miR‐149‐5p. LncRNA MALAT1 was observed to act as a sponge for miR‐206, so as to promote trophoblast cells migration and invasion.^20^ However, the competitive endogenous RNA mechanism of lncRNA‐ATB in preeclampsia has not been reported.

MiRNAs are non‐coding RNAs containing 19‐22 nucleotide sequences that act as regulators of endogenous epigenetic gene expression. They are transcribed by RNA polymerases II and III into large primary miRNAs or pre‐miRNAs, which are then treated by the ribonucleases Drosha and Dicer to form mature miRNAs. These mature miRNAs are then integrated into the RISC and bind to the 3′‐UTR mRNA complementary region, inducing degradation or inhibiting translation, resulting in gene silencing.^21^ Maladjustment miRNAs in placenta or plasma of PE patients are involved in trophoblast invasion, vascular endothelial injury, and other pathological processes through many targeted genes, promoting or inhibiting the occurrence and development of PE. For example, Yu et al^22^ found that miR‐204 may be involved in the occurrence of PE by inhibiting trophoblast invasion, while MMP9 may play a role as a target gene for miR‐204. Huang et al^23^ indicated that miR‐139‐5p was down‐regulated in severe PE patients and was negatively correlated with the expression of sFlt‐1, suggesting a positive effect on the proliferation and invasion of trophoblast cells by directly targeting sFlt‐1 in PE. Moreover, we recently published a meta‐analysis on the diagnostic and predictive value of maternal circulating miRNAs for PE.^24^ The results of the quantitative summary showed that circulating miRNAs could distinguish between PE patients and control groups, and had high accuracy in diagnosis and prediction. We then concluded that circulating miRNAs may be a useful screening tool for the diagnosis and prediction of PE. In summary, miRNAs associated with PE have been reported in several studies. However, miR‐651‐3p has not been associated with any disease and for the first time we investigated its mechanism of action in PE.

In this study, we discussed YY1 as the target gene for miR‐651‐3p. YY1, known as Yin Yang 1, is a widely distributed transcription factor belonging to GLI‐Krüppel zinc finger protein. Yin Yang 1 plays a fundamental role in normal biological processes, such as embryogenesis, differentiation, replication and cell proliferation. Yin Yang 1 acts on genes involved in these processes through its ability to initiate, activate, or inhibit transcription.^25^ In recent years, many studies have found that non‐coding RNAs can be regulated by YY1 or act as the regulator of YY1.^26^ Tians et al^27^, ^28^ published two articles that explored the regulatory pathway of YY1 in trophoblast invasion of early pregnancy, suggesting that YY1 may be involved in the pathogenesis of recurrent miscarriage. However, YY1 has not been studied in the pathogenesis of PE.

In the present study, we validated that lncRNA‐ATB was lowly expressed in placental tissues of PE patients and then we constructed a co‐culture model of spiral artery remodelling and explored the effects of the lncRNA‐ATB/miR‐651‐3p/YY1 pathways on the model.

## 2 MATERIALS AND METHODS

### 2.1 Placenta sample collection and clinical features

We collected placenta samples from pregnant women diagnosed with PE (n = 24) and normal pregnant women (n = 26) who underwent cesarean section at West China Second Hospital of Sichuan University from 2019 to 2020. Samples were taken within 30 minutes after surgery and a small piece of the maternal surface about 1 cm^3^ was taken and washed with ice phosphate buffer saline (PBS) and immediately placed in a −150°C refrigerator for preservation. This study was approved by the Ethics Committee of West China Second Hospital and all participants in the study signed informed consent forms.

Preeclampsia is defined as SBP of 140 mm Hg or greater or DBP of 90 mm Hg or greater on two occasions at least 4 hours apart after 20 weeks of gestation in a woman with a previously normal blood pressure. In addition, abnormal blood pressure may be accompanied by proteinuria that is greater than 0.3 g per day or absence of proteinuria but one of the following emerging symptoms: thrombocytopenia, renal insufficiency, impaired liver function, pulmonary edema or new‐onset headache. Patients with diabetes, heart disease, chronic hypertension, chronic nephritis, autoimmune disease, thrombotic disease, HELLP syndrome and infectious disease were excluded from the study.

### 2.2 Cell culture

Neonatal umbilical cords were collected from the obstetrics department of West China Second Hospital. Healthy pregnant women without complications were selected, and about 20 cm neonatal umbilical cord was obtained by aseptic operation after cesarean section. Human umbilical vein endothelial cells were digested using Collagenase type 1 (Sigma) and then cultured in HUVEC complete medium (Cyagen) supplemented with of 10% fetal bovine serum (FBS), 1% penicillin‐streptomycin, 1% glutamine, 1% endothelial cell growth supplement, and 1% heparin. Phycoerythrin (PE) anti‐human CD31 antibody (Biolegend) was detected using flow cytometry (FACSCelesta, BD Biosciences) and the purity of HUVECs was determined. Subsequent analyses were carried out when the purity of HUVEC was greater than 90%. Only three to six generations were used for the study. The human first‐trimester extravillous trophoblast cell line HTR8/SVneo cells were generously donated by Professor Yali Hu from affiliated Drum Tower Hospital, Medical College of Nanjing University, Nanjing, China. These trophoblast cells were cultured in RPMI 1640 medium (HyClone) supplemented with 10% FBS and 1% penicillin‐streptomycin. HEK‐293T cells were purchased from Chinese Academy of Sciences (Shanghai, China), and cultured in Dulbecco's Modified Eagle Medium (DMEM, HyClone) supplemented with 10% FBS and 1% penicillin‐streptomycin. Both of later cells were identified by Short Tandem Repeat (STR). All cells were cultured at 37°C with 5% CO_2_.

### 2.3 Co‐culture of endothelial cells and trophoblast cells

Glass bottom (10 mm) confocal culture dishes (Jing'an, China) were coated with 80 μL Matrigel (BD Biosciences) and incubated for 30 minutes to solidify. HUVEC (2 × 10^4^) cells stained with 1 μmol/L CellTracker^™^ Green CMFDA Dye (Thermo FisherScientific) were then added into the Matrigel‐coated dishes. After 4 hours, HUVECs formed tubular structures, then the medium was removed and carefully washed with PBS. Thereafter, 2 × 10^4^ treated HTR8/SVneo cells that were stained with 1 μmol/L CellTracker^™^ Red CMTPX Dye (Thermo Fisher Scientific) were added to the HUVEC layer. After co‐culture for 4 hours, the medium was removed and fixed with 4% paraformaldehyde for 30 minutes. Images of random regions were taken from each dish using a confocal microscope (Olympus FV1000). Quantification was performed using the Image J software (National Institutes of Health). Images were converted to 8‐bit gray scale with a defined preset threshold. The tubular area was then calculated as the number of pixels. The percentage of HTR8/SVneo in the tube green fluorescence area/red fluorescence area.

### 2.4 Scratch wound healing assay

While transfected for 24 hours and confluent to 90%, the monolayer cells were scratched by a 10 μL pipette tip in a straight line and washed with PBS subsequently. Then, the cells were cultured in serum‐free medium for 24 hours. The percentage of wound closure was measured using Image J.

### 2.5 Cell invasion assay

The Matrigel (Corning) was diluted with serum‐free RPMI 1640 medium at 1:40. The upper transwell chamber (Corning) was added with 100 μL diluted Matrigel, and placed at 37°C for 3 hours for later use. 10^5^ transfected HTR8/SVneo cells in 200 μL serum‐free RPMI 1640 medium were added to the upper chamber and 700 μL of complete medium were added to the lower chamber. After cultivation for 24 hours, the invading cells on the surface of inserts bottom were fixed with 4% paraformaldehyde for 30 minutes and stained with 0.1% crystal violet for 5 minutes. The cells inside the upper chamber were carefully wiped out with a cotton swab. Finally, the number of invaded cells was calculated with Image J.

### 2.6 RNA extraction and quantitative real‐time PCR

Total RNA was extracted from placental samples and the treated cells using the chloroform‐free RNA extraction kit (Bioteke). The concentration and purity of nucleic acid were determined using nanophotometer (Implen) within a controllable range before further experiments. Complementary DNA (cDNA) synthesis for LncRNA, miRNA and mRNA was carried out using the PrimeScript^™^ RT reagent Kit (Takara). However, miRNA cDNA synthesis used special reverse transcription primers designed by the stem‐loop method as follows: miR‐651‐3p, 5′‐GTCGTATCCAGTGCGTGTCGTGGAGTCGGCAATTGCACTGGATACGACCTTTTAGG‐3′. The SYBR^®^ Green Realtime PCR Master Mix was used to conduct quantitative real‐time PCR (qRT‐PCR) reaction on the CFX96 real‐time PCR detection system (BioRad). The relatively expression levels of lncRNA‐ATB and YY1 were normalized using β‐actin, while those ford miR‐651‐3p were normalized using U6 small nuclear B non‐coding RNA (U6). The relative expression levels were analyzed using the 2^−ΔΔCt^ method. The primers for qRT‐PCR were as follows:

**Table:** 

lncRNA‐ATB forward	5′‐TGGGATTCGATCAACAGAGAGT‐3′
lncRNA‐ATB reverse	5′‐CATACTGCCCCTCCCGTTTG‐3′
miR‐651‐3p forward	5′‐GCGCAAAGGAAAGTGTATCC‐3′
miR‐651‐3p reverse	5′‐CAGTGCGTGTCGTGGAGT‐3′
YY1 forward	5′‐AAGAGCGGCAAGAAGAGTTAC‐3′
YY1 reverse	5′‐CAACCACTGTCTCATGGTCAATA‐3′
β‐actin forward	5′‐CATGTACGTTGCTATCCAGGC‐3′
β‐actin reverse	5′‐CTCCTTAATGTCACGCACGAT‐3′
U6 forward	5′‐CTCGCTTCGGCAGCACA‐3′
U6 reverse	5′‐AACGCTTCACGAATTTGCGT‐3′

### 2.7 Transfection and co‐transfection

The lncRNA‐ATB small interference RNA (si‐ATB), YY1 siRNA (si‐YY1) and their corresponding negative control siRNA (si‐NC), miR‐651‐3p mimics and mimics NC, miR‐651‐3p inhibitor and inhibitor NC were designed using Primer‐BLAST (https://www.ncbi.nlm.nih.gov/tools/primer‐blast/) and synthesized using RiboBio. The lncRNA‐ATB overexpression pcDNA3.1(+) vector (pcDNA3.1‐ATB), YY1 overexpression pcDNA3.1(+) vector (pcDNA3.1‐YY1) and empty vector (pcDNA3.1) were synthesized by Tsingke. Transfection was performed in six‐well plates when cells confluency was 50%‐70% using Lipofectamine^™^ 3000 Transfection Reagent (Invitrogen). Co‐transfection assays were used as rescue experiments to better demonstrate the interaction between the two molecules. pcDNA3.1‐ATB or pcDNA3.1‐YY1 and mimics or mimics NC were mixed first, and then transfected into the cells. Further assays were carried out from 24 to 48 hours after transfection.

### 2.8 Dual‐luciferase reporter assay

Since few databases can predict lncRNA‐binding miRNAs, we only used miRDB (http://mirdb.org/custom.html) to predict miRNAs that might bind to lncRNA‐ATB. On the other hand, miRDB, Targetscan (http://www.targetscan.org/vert_72/), Diana (http://carolina.imis.athena‐innovation.gr/diana_tools/web/index.php?r=lncbasev2/index‐predicted) and Mirtarbase (http://mirtarbase.cuhk.edu.cn/php/search.php) were used to predict potential mRNA targets for the miRNAs. Potential target sequences for miR‐651‐3p in the lncRNA‐ATB and YY1 3′‐UTR were synthesized into the pSI‐Check2 plasmid by Hanbio to form the wild type dual‐luciferase reporter plasmid (WT) or the mutant type (MUT). HEK293T cells were seeded in 96‐well plates and co‐transfected with plasmids and mimics or mimics NC using lipo3000. After 24 hours transfection, chemiluminescence intensity was measured using the Dual Glo^®^ Luciferase Assay System (E2920, Promega) using a microplate reader (infiniteM200, Tecan).

### 2.9 Western blot analysis

Proteins from tissue and cell lines were extracted using a total protein extraction kit (BestBio). The protein concentration was determined using Micro BCA^™^ Protein Assay Kit (Thermo Fisher Scientific) and after incubation for 1 hour, the protein concentration of samples and standards was determined at a wavelength of 562 nm using a microplate reader. All proteins were standardized to a concentration of 3 μg/μL and denatured at 99°C for 5 minutes. Total proteins were separated using TGX Stain‐Free^™^ FastCast^™^ Acrylamide Kit (BioRad) and transferred to the polyvinylidene difluoride membranes (PVDF, 16 20 177, BioRad). Subsequently, the membranes were blocked with 5% Bovine Serum Albumin (BSA) for 3 hours and incubated with primary antibody YY1 (1:12 000, Abcam) and glyceraldehyde‐3‐phosphate dehydrogenase (GAPDH, 1:5000, ZenBio) overnight at 4°C. The membranes were washed three times with TBST and then incubated with horseradish peroxidase (HRP)‐conjugated secondary antibody goat anti‐rabbit (1:10 000, HuaBio) and goat anti‐mouse (1:2000, HuaBio) for 1 hour at room temperature. Lastly, Immobilon Western Chemiluminescent HRP Substrate (Millipore) was used to detect the chemiluminescence intensity via the ChemiDoc^™^ MP Imaging System (BioRad).

### 2.10 Statistical analysis

All the statistical computations were performed using GraphPad Prism version 5 software (GraphPad). First, the data were tested for normality and homogeneity of variance. Data that conformed to the normal distribution, was presented as mean ± standard deviation (SD), otherwise it was presented by median and interquartile range (IQR). For quantitative data, *t* test/ANOVA/rank sum test were used for comparison. The bivariate correlation was analyzed using Pearson correlation/Spearman rank correlation analysis. *P* < .05 was considered to be statistically significant.

## 3 RESULTS

### 3.1 Establishment of co‐culture model

**FIGURE 1:**
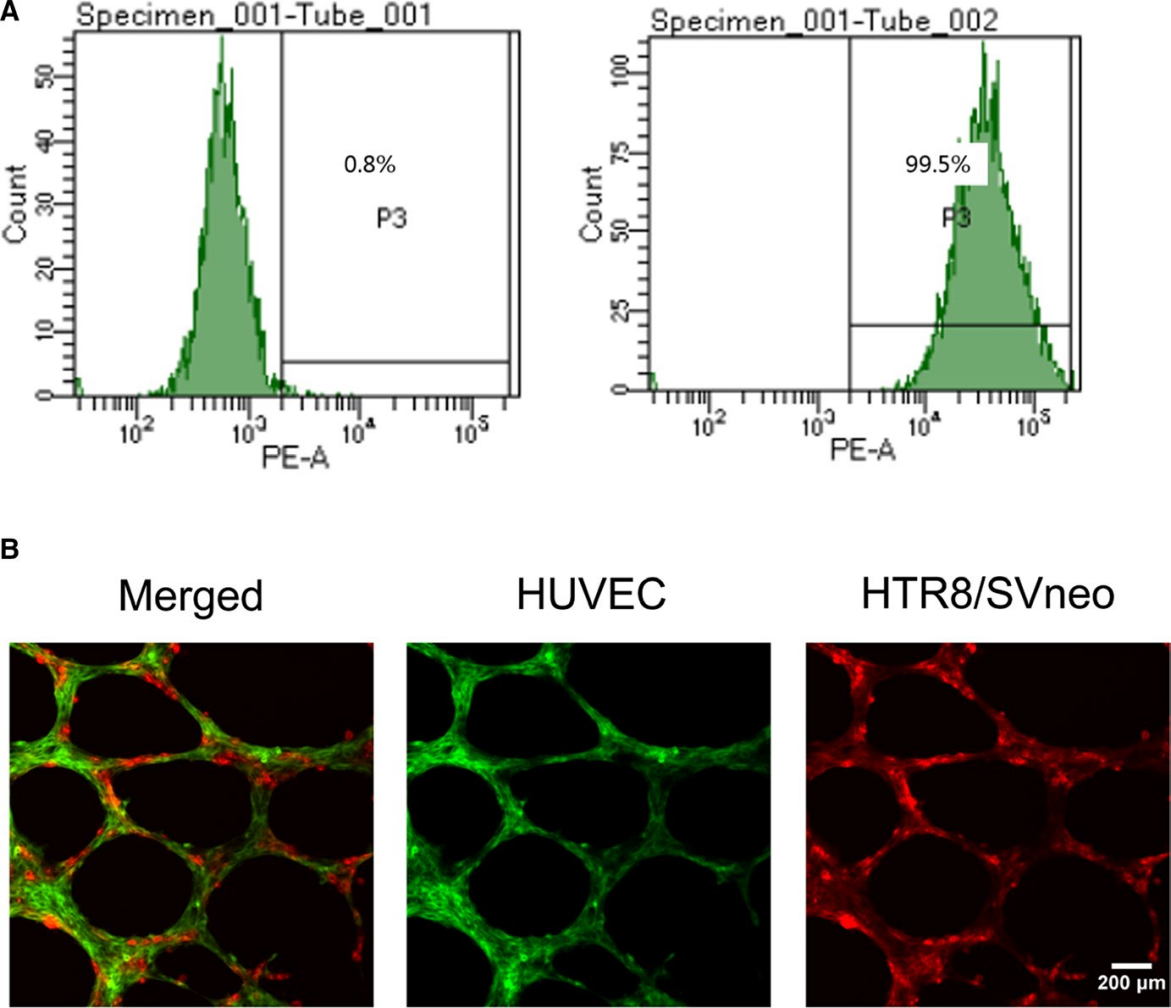
CD31 expression of human umbilical vein endothelial cells detected by flow cytometry and representative images of co‐cultures networks. A, The expression level of CD31 antigen was 0.8% in the control group, while it was 99.5% in the experimental group. B, The representative images showed that HTR8/SVneo cells integrated into endothelial cellular networks. Original magnification ×200. The scale bars indicate 200 μm

Human umbilical vein endothelial cells were extracted from the umbilical cord of cesarean section women with normal pregnancy. Flow cytometry analysis showed that only 0.8% of the cells in the negative control group expressed CD31 protein, while the expression level of CD31 protein in the experimental group was 99.5%, indicating the successful extraction of HUVECs (Figure 1A). An in vitro co‐culture model was established to simulate arterial remodelling process in vivo. On the existing HUVEC tubular structure, trophoblast cells were actively moving towards to the HUVEC tubular structure and were fully connected and replaced (Figure 1B).

### 3.2 LncRNA‐ATB is downregulated in PE tissues

**FIGURE 2:**
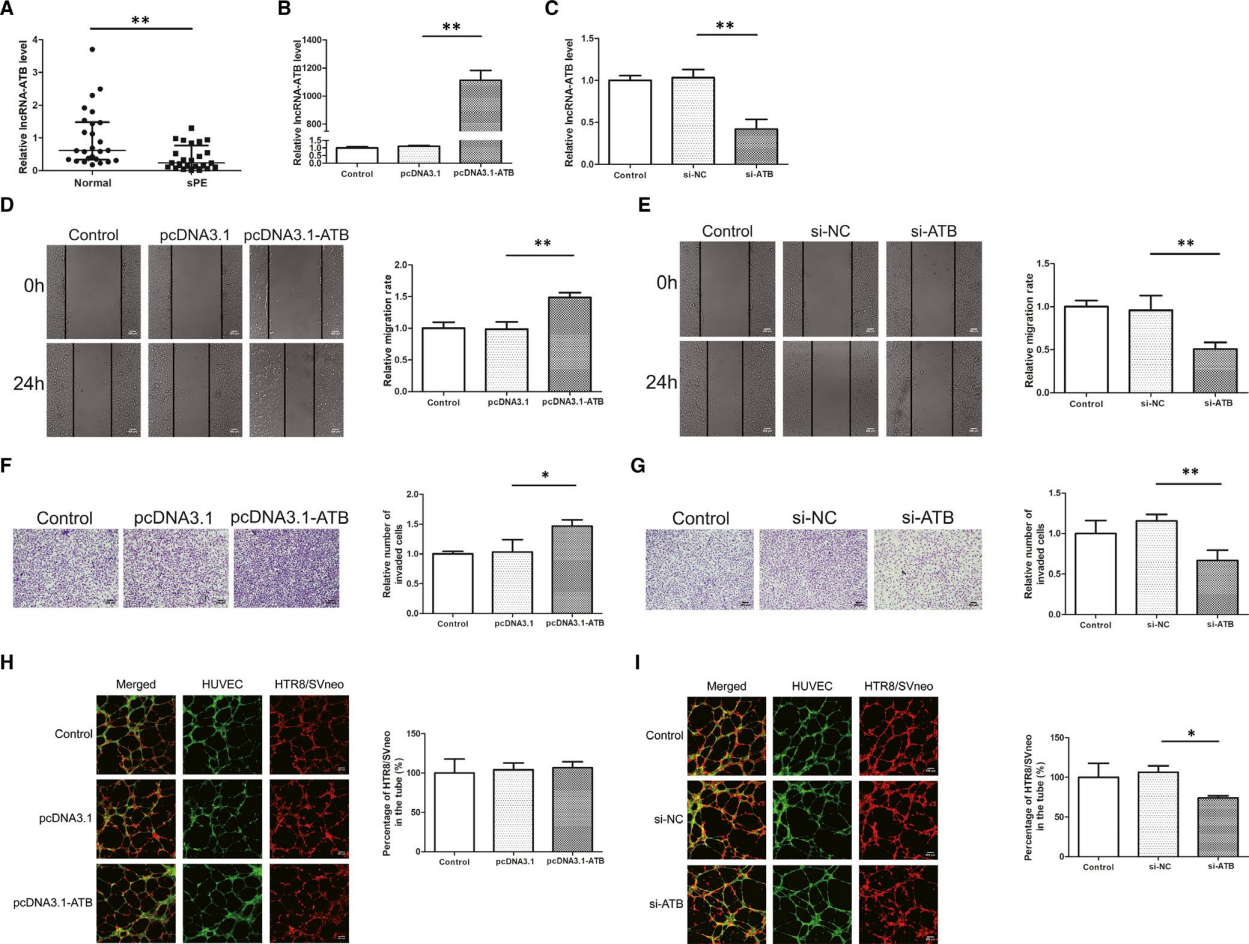
Expression of lncRNA‐ATB in PE tissues and effects on cell migration, invasion, and co‐culture model. A, The quantitative real‐time PCR results showed that the expression of placental lncRNA‐ATB in PE patients was lower than that in normal pregnant women. B, C, The expression of lncRNA‐ATB was significantly increased in HTR8/SVneo transfected with pcDNA3.1‐ATB and decreased with si‐ATB compared to control groups. D, E, Overexpression of lncRNA‐ATB in HTR8/SVneo cells resulted in increasing wound closure ability and lncRNA‐ATB knockdown reduced the rate of wound closure. Original magnification×100. The scale bars indicate 500 μm. F, G, Overexpression of lncRNA‐ATB significantly increased cell invasion while knockdown of lncRNA‐ATB reduced cell invasion compared with the control group. Original magnification×100. The scale bars indicate 200 μm. H, I, HTR8/SVneo cell integration into endothelial cellular networks was significantly inhibited by si‐ATB treatment while lncRNA‐ATB overexpression did not affect the integration. Original magnification×100. The scale bars indicate 500 μm. *P < .05, **P < .01

Placental specimens from 26 normal pregnancies and 24 PE pregnancies were collected and the clinical features are shown in Table 1. Analysis of the clinical features showed that there was no difference in maternal age while there was a significant difference in gestational age and nulliparity between the two groups. In addition, the PE group had higher SBP, DBP, and proteinuria which correlated with the diagnostic criteria for PE. In terms of fetal outcomes, fetal weight and fetal length were lower in the PE group than in the control group. No statistical difference was seen in fetal sex between the two groups. The qRT‐PCR results showed that the expression of placental lncRNA‐ATB in PE patients was lower than that in normal pregnant women, which was consistent with our previous study (Figure 2A).

**TABLE 1:** Clinical characteristics of normal and preeclamptic pregnancies

	Control group (n = 26)	PE group (n = 24)
Maternal age (y)	32.50 ± 3.23	30.71 ± 4.94
Gestational age (wk)	38.47 ± 0.87	35.38 ± 2.32***
BMI (kg/m^2^)	25.64 ± 2.54	27.81 ± 3.10**
Nulliparity (%)	42.31%	75%*
SBP (mm Hg)	117.00 ± 11.08	148.0(145.0‐157.0)***
DBP (mm Hg)	73.77 ± 8.77	99.38 ± 7.93***
Proteinuria (g/24h)	0	3.04(1.26‐6.72)***
Birth weight (g)	3339 ± 455.1	2176 ± 662.0***
Birth length (cm)	49.00(49.00‐50.25)	43.50 ± 4.89***
Baby's sex (%; male)	61.54%	45.83%

Note

Results are presented as mean ± standard deviation or median (interquartile range; *P <.05, **P <.01, ***P <.001 by unpaired *t* test or nonparametric test).

Abbreviations: BMI, body mass index; DBP, diastolic pressure; PE, preeclampsia; SBP, systolic pressure.

### 3.3 Effects of lncRNA‐ATB on migration, invasion of trophoblast cells and trophoblast‐endothelial cell interactions networks

To investigate the effects of lncRNA‐ATB on the migration, invasion of trophoblast cells and interaction between trophoblast cells and endothelial cells, lncRNA‐ATB was either overexpressed or silenced in HTR8/SVneo. The qRT‐PCR results showed that the expression of lncRNA‐ATB was significantly increased in HTR8/SVneo transfected with pcDNA3.1‐ATB (Figure 2B). Meanwhile, the cells transfected with siRNA‐ATB had lower expression compared to si‐NC (Figure 2C). As expected, wound healing assay suggested that lncRNA‐ATB overexpression promoted cell migration while lncRNA‐ATB knockdown inhibited cell migration (Figure 2D,E). In addition, transwell assay results showed that overexpression of lncRNA‐ATB would increase the number of invaded cells, while knockdown of lncRNA‐ATB significantly reduced it (Figure 2F,G). LncRNA‐ATB overexpression did not affect the integration of the two cells, however, si‐ATB treatment resulted in reduction of HTR8/SVneo which invaded into HUVEC tubular structures compared to the control group (Figure 2H,I).

### 3.4 LncRNA‐ATB acted as a sponge of miR‐651‐3p in trophoblast cells

**FIGURE 3:**
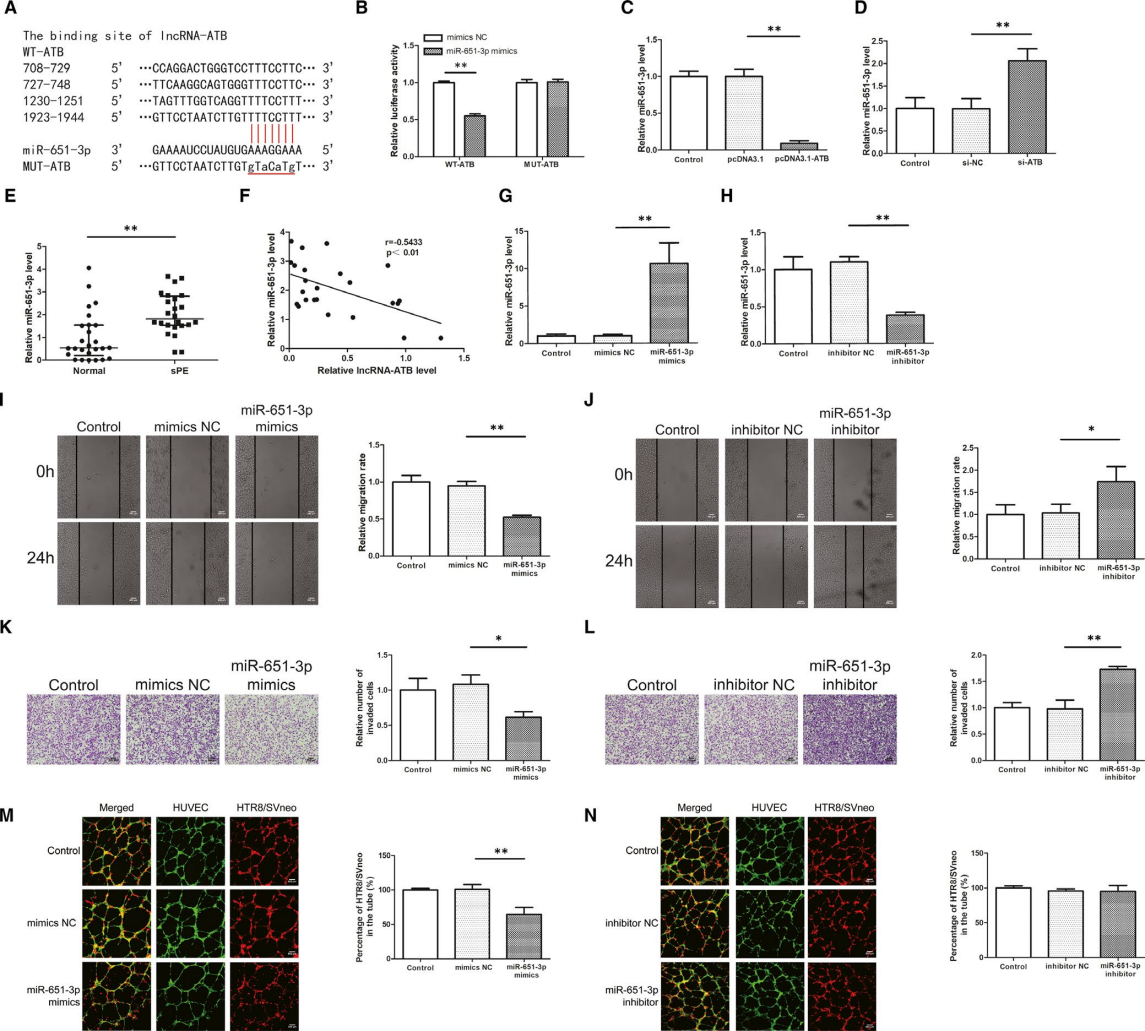
MiR‐651‐3p is a target of lncRNA‐ATB and its role on migration, invasion and co‐culture model. A, The binding sites between lncRNA‐ATB and miR‐651‐3p were predicted by miRDB. B, The results showed that luciferase activity was decreased in 293T cells co‐transfected with WT‐ATB plasmids and miR‐651‐3p mimics compared to the mimics NC group, whereas the luciferase activity in the MUT‐ATB group remained unchanged. C, D, MiR‐651‐3p expression was detected in cells transfected with pcDNA3.1‐ATB or si‐ATB by quantitative real‐time PCR analysis. E, The expression of miR‐651‐3p in the placenta was higher in PE than the normal pregnancies. F, MiR‐651‐3p was inversely correlated with the expression of lncRNA‐ATB assessed by Pearson analysis (*r *= −.5431, *P* <.01). G, H, The successful transfection of miR‐651‐3p mimics and inhibitor led to the overexpression and low‐expression of miR‐651‐3p in HTR8/SVneo cells. I, J, Scratch wound assay was conducted to measure cell migration. K, L, Tanswell assay was used to detect the ability of invasion. M, N, Co‐culture assay was performed to test the ability of trophoblast cells to co‐locate to endothelial cells networks. *P <.05, **P <.01

**FIGURE 4:**
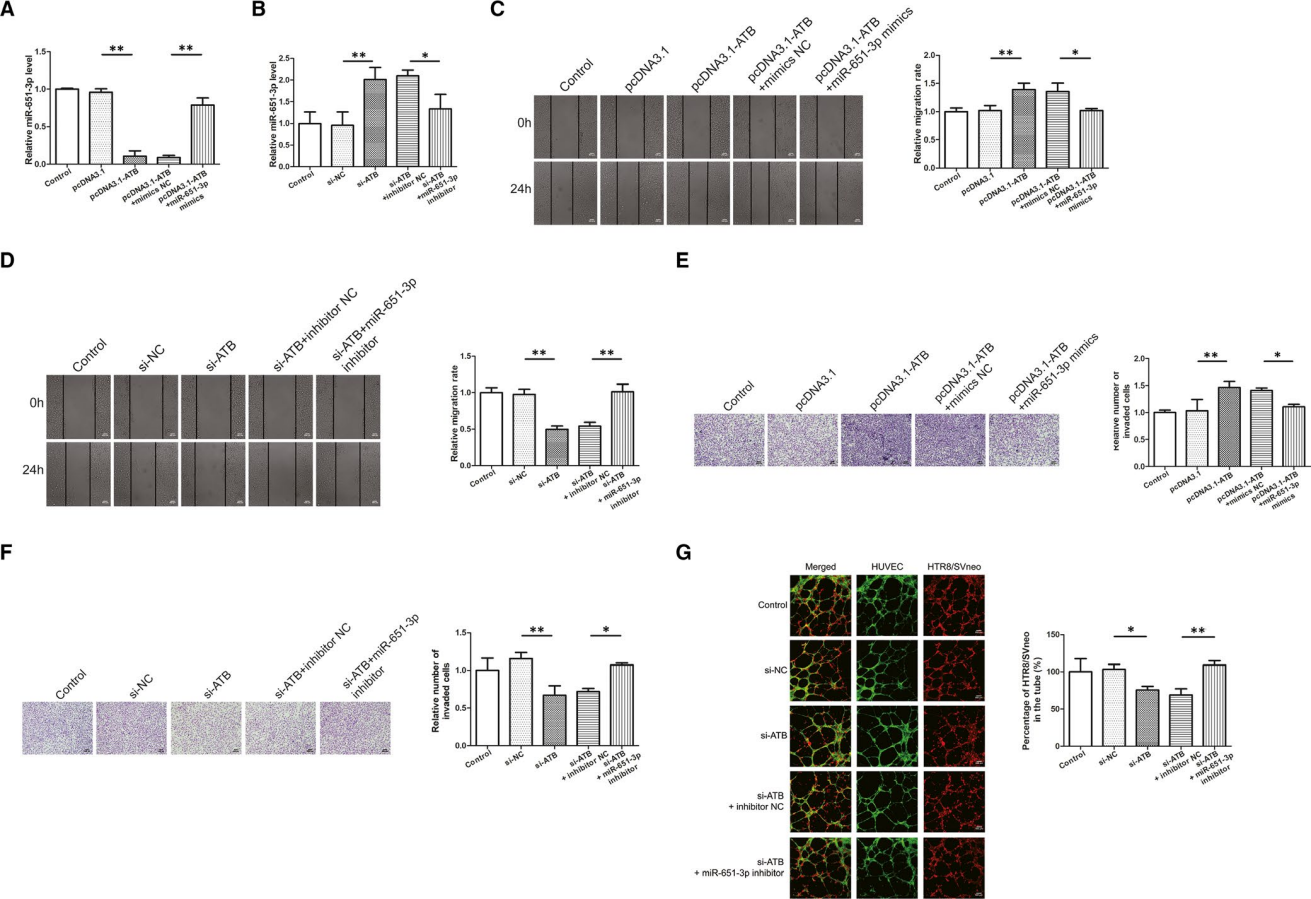
LncRNA‐ATB modulates cell migration, invasion, and trophoblast‐endothelial cell interactions via sponging miR‐651‐3p. A, B, The expression level of miR‐651‐3p was detected by quantitative real‐time PCR after co‐transfection. C, D, Cell migration was detected by wound healing assay. E, F, Cell invasion was measured by transwell assay. G, Co‐culture experiment revealed that lncRNA‐ATB knockdown inhibited the colocalisation, while miR‐651‐3p silencing relieved the inhibition. *P <.05, **P <.01

First, potential target miRNA of lncRNA was predicted using miRDB and the specific wild and mutant type sequences are shown in Figure 3A. Dual‐luciferase reporter assay was then used to verify this prediction. The results showed that luciferase activity was decreased in 293T cells co‐transfected with WT‐ATB plasmids and miR‐651‐3p mimics compared to the mimics NC group, whereas the luciferase activity in the MUT‐ATB group remained unchanged (Figure 3B). In addition, qRT‐PCR results showed that overexpression of lncRNA‐ATB led to a decrease in miR‐651‐3p expression while transfection with si‐ATB led to an increase in miR‐651‐3p expression (Figure 3C,D). The expression of miR‐651‐3p in the placenta was higher in PE than the normal pregnancies and inversely correlated with the expression of lncRNA‐ATB (Figure 3E,F). In order to further verify the role of miR‐651‐3p in PE, the transfection efficiency of the miR‐651‐3p mimics and inhibitor were determined to meet the requirements in HTR8/SVneo cells (Figure 3G,H). With wound scratch and cell invasion assay, we found that miR‐651‐3p mimics deceased the migration and invasion ability of trophoblasts cells and miR‐651‐3p inhibitor led to an increase (Figure 3I‐L). Moreover, the down regulation of miR‐651‐3p resulted in a decrease in the number of trophoblast and endothelial cells co‐localisation, while the overexpression did not show any difference from the control group (Figure 3 M,N). Then, lncRNA‐ATB and miR‐651‐3p were overexpressed or knocked down simultaneously, and it was found that miR‐651‐3p expression was rescued by pcDNA3.1‐ATB or si‐ATB on the basis of transfected with mimics or inhibitor (Figure 4A,B). The results showed that overexpression of lncRNA‐ATB enhanced the ability of migration and invasion, but the simultaneous up‐regulation of miR‐651‐3p weakened these abilities (Figure 4C,E). Lower migration and invasion abilities were observed in the si‐ATB group compared with the control group, while the decline of these abilities was prevented by co‐transfection with miR‐651‐3p inhibitor (Figure 4D,F). Co‐culture experiment revealed that lncRNA‐ATB knockdown inhibited the colocalisation, while miR‐651‐3p silencing relieved the inhibition (Figure 4G).

### 3.5 Yin Yang 1 was a target gene of miR‐651‐3p in trophoblast cells

**FIGURE 5:**
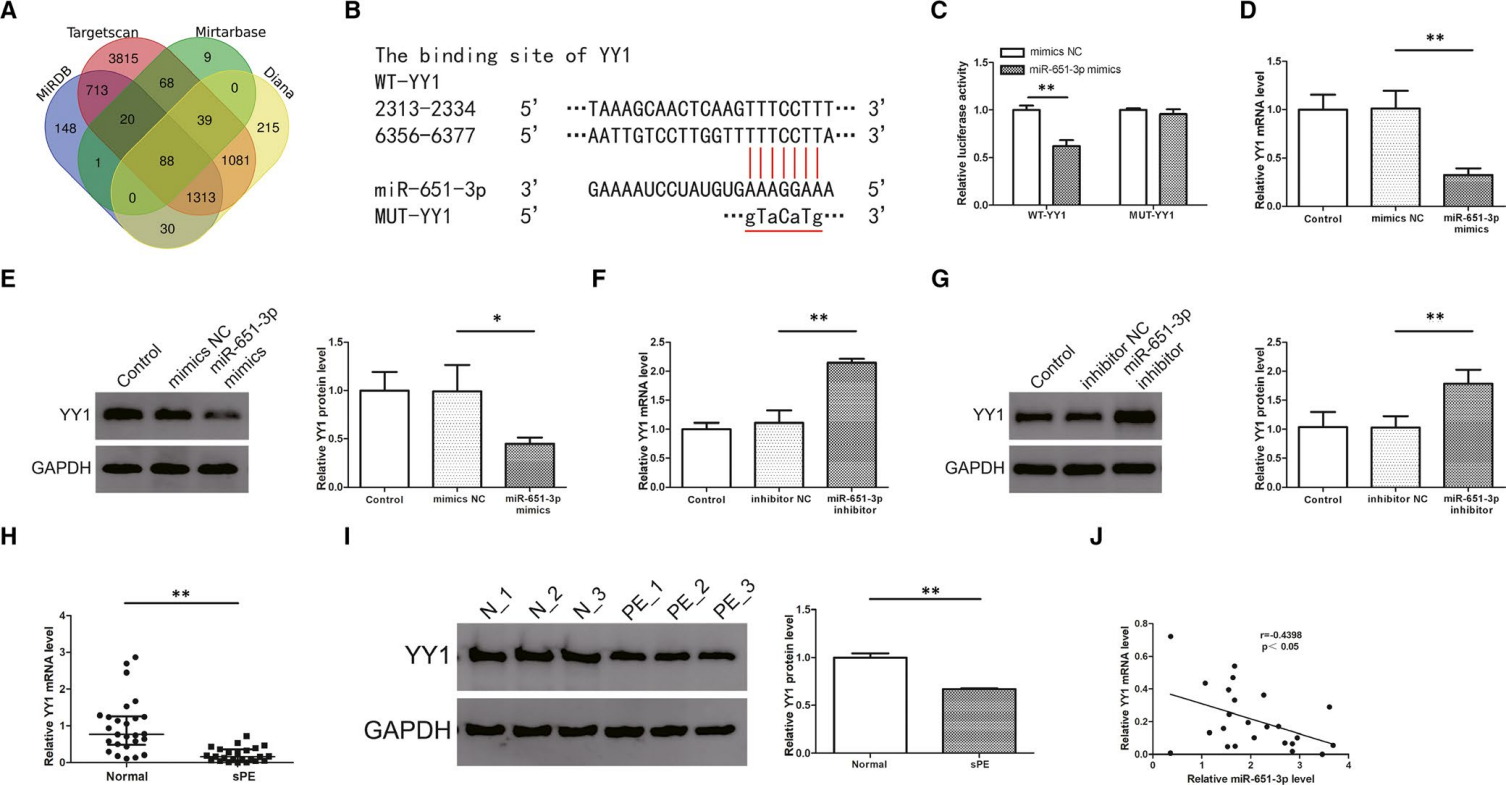
Yin Yang 1 (YY1) was the target gene of miR‐651‐3p in trophoblast cells. A, The Venn diagram showed that four databases identified 88 potential target genes for miR‐651‐3p. B, The binding sites between YY1 and miR‐651‐3p were predicted by miRDB, Mirtarbase, Targetscan, Diana. C, Dual‐luciferase reporter assay performed in 293T cells showed that there was a significant reduction in luciferase activity in cells co‐transfected with miR‐651‐3p and YY1‐WT compared to cells co‐transfected with miR‐651‐3p and YY1‐MUT. D‐G, Transfection of HTR8/SVneo with miR‐651‐3p led to a decrease in the mRNA and protein levels of YY1 while transfection with miR‐651‐3p inhibitor led to an increase in the levels. H, I, The mRNA and protein expression levels of placental YY1 in PE patients were lower than that in the control group. (Data are showed as median and IQR. Statistical analysis was performed with nonparametric test.) J, MiR‐651‐3p was inversely correlated with the expression of lncRNA‐ATB assessed by Pearson analysis (*r *= −.4398, *P* <.05). *P <.05, **P <.01

Four databases identified 88 potential target genes for miR‐651‐3p (Figure 5A). We singled out 10 candidate genes that had previously been associated with various diseases. Expression analysis using qRT‐PCR revealed that only YY1 mRNA expression was reduced in miR‐651‐3p mimics transfected HTR8/SVneo cells. The possible binding sites of miR‐651‐3p on YY1 are shown in Figure 5B. Results of dual‐luciferase reporter assay performed in 293T cells showed that there was a significant reduction in luciferase activity in cells co‐transfected with miR‐651‐3p and YY1‐WT compared to cells co‐transfected with miR‐651‐3p and YY1‐MUT (Figure 5C). In addition, transfection of HTR8/SVneo with miR‐651‐3p led to a decrease in the mRNA and protein levels of YY1 while transfection with miR‐651‐3p inhibitor led to an increase in the levels (Figure 5D‐G). The mRNA and protein expression levels of placental YY1 in PE patients were lower than that in the control group (Figure 5H,I). Moreover, the mRNA expression level of YY1 in the PE group was inversely correlated with miR‐651‐3p (Figure 5J).

### 3.6 Effect of Yin Yang 1 on trophoblast‐endothelial cell interactions networks was mediated by miR‐651‐3p

**FIGURE 6:**
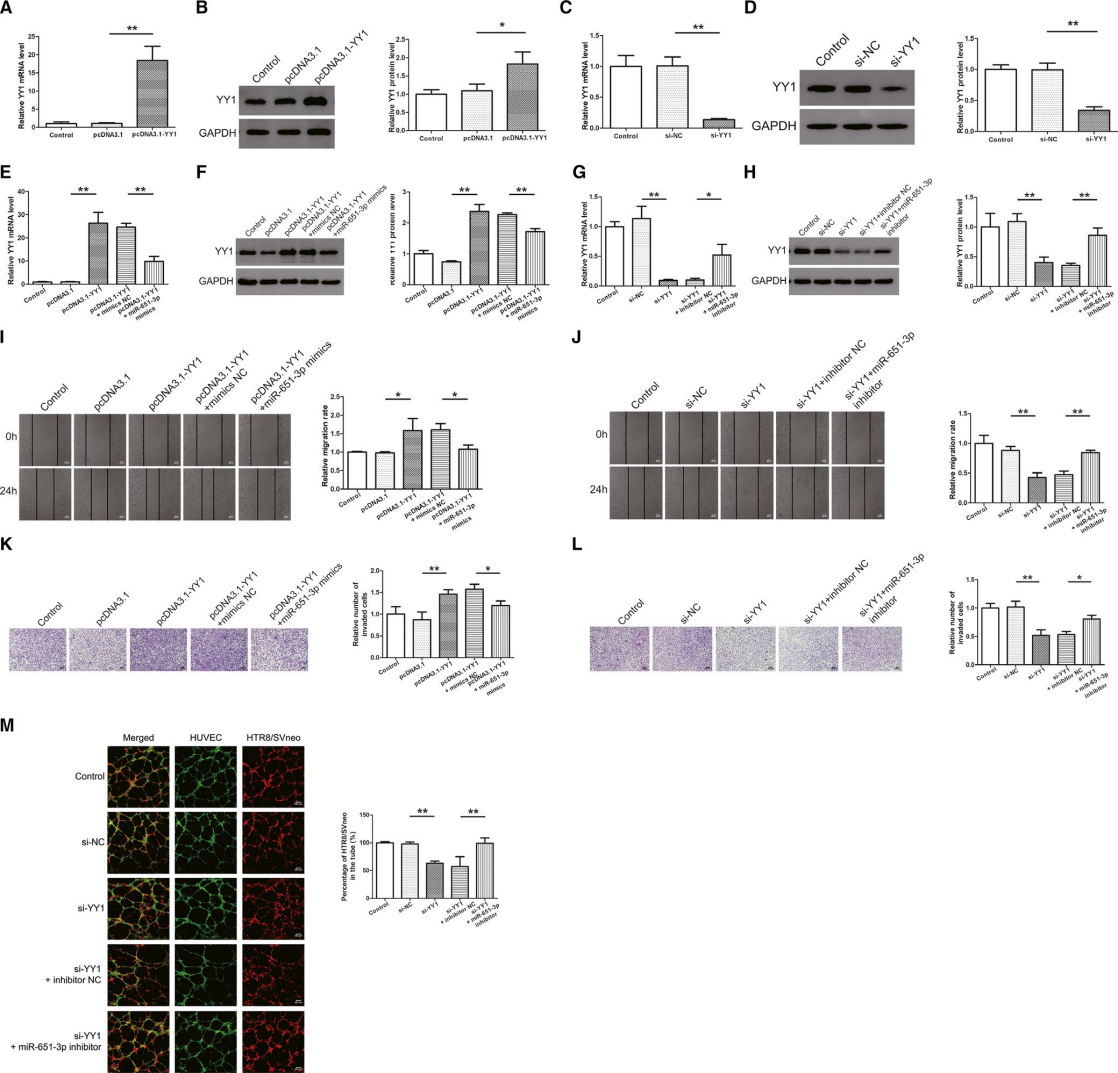
Effect of Yin Yang 1 (YY1) on cell migration, invasion and trophoblast‐endothelial cell interactions networks was mediated by miR‐651‐3p. A‐D, The quantitative real‐time PCR and western blot results showed that pcDNA3.1‐YY1 increased, while siRNA‐YY1 decreased the mRNA and protein expression levels of YY1. E‐H, Co‐transfection experiments of miR‐651‐3p and YY1 was conducted to further verify the direct effects. I, J, Cell migration was detected by wound healing assay. K, L, Cell invasion was measured by transwell assay. M, Si‐YY1 significantly decreased HTR‐8/SVneo cells integration into endothelial cellular networks whereas the effect was reversed by co‐transfection with miR‐651‐3p inhibitor. *P <.05, **P <.01

First, the transfection efficiency was determined in HTR8/SVneo cells and the results showed that pcDNA3.1‐YY1 increased, while siRNA‐YY1 decreased the mRNA and protein expression levels of YY1 (Figure 6A‐D). Co‐transfection experiments of miR‐651‐3p and YY1 was conducted to further verify their direct effects (Figure 6E‐H). As the results revealed that overexpression of YY1 promoted the migration and invasion of trophoblast cells, and miR‐651‐3p mimics could reverse that promotion (Figure 6I,K). Lower migration and invasion abilities were observed in the si‐YY1 group compared with the control group, while the decline of these abilities was prevented by co‐transfection with miR‐651‐3p inhibitor (Figure 6J,L). As for co‐culture experiment, si‐YY1 significantly decreased HTR‐8/SVneo cells integration into endothelial cellular networks whereas the effect was reversed by co‐transfection with miR‐651‐3p inhibitor (Figure 6M).

### 3.7 LncRNA‐ATB affected YY1 expression by sponging miR‐651‐3p

**FIGURE 7:**
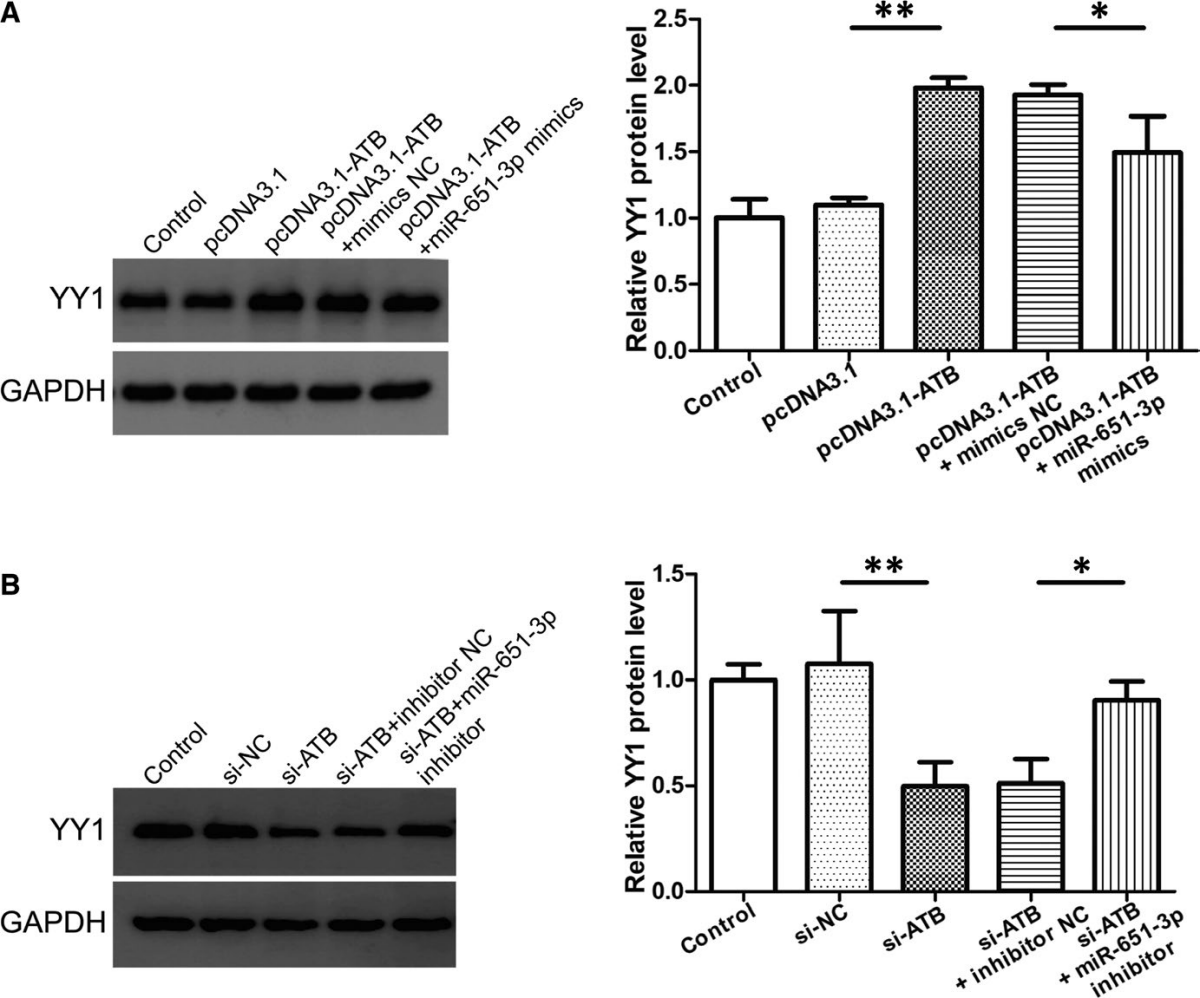
LncRNA‐ATB affected Yin Yang 1 (YY1) expression by sponging miR‐651‐3p. A, B, Western blot analysis showed that miR‐651‐3p mimics reversed the pcDNA3.1‐ATB‐induced increase in YY1 expression, and miR‐651‐3p inhibitor reversed the si‐ATB‐induced decrease in YY1 expression. *P <.05, **P <.01

LncRNA‐ATB and miR‐651‐3p were overexpressed or silenced simultaneously. Western blot analysis showed that miR‐651‐3p mimics reversed the pcDNA3.1‐ATB‐induced increase in YY1 expression, and miR‐651‐3p inhibitor reversed the si‐ATB‐induced decrease in YY1 expression (Figure 7A,B). In general, our results showed that lncRNA‐ATB regulated the expression of YY1 by acting as a sponge of miR‐651‐3p, thus regulating the spiral artery remodelling process.

## 4 DISCUSSION

Preeclampsia is a serious complication of pregnancy and a cause of maternal mortality. Although the etiology of PE remains controversial, clinical and pathological studies suggest that the placenta is central to the pathogenesis of this syndrome.^29^ Therefore, termination of pregnancy, namely placenta removal, is the only effective and definite treatment. Defective deep placentation was first described in PE and intrauterine growth restriction (IUGR) and was characterized by absent or incomplete remodelling of the junctional zone segment of the spiral arteries.^30^ In this study, we wanted to simulate the spiral artery remodelling in vitro with Matrigel that was better than the established biological behaviour, such as invasion, migration and tube formation. It has been reported in several studies that the co‐culture model of endothelial cells and the other cells associated with diseases can simulate the phenotypic and morphological characteristics in vivo. Baimakhanov et al^31^ observed that primary hepatocytes migrated spontaneously to the HUVEC tube network, and they hypothesized that this co‐culture model duplicated part of their normal physiology. In this study, HUVEC was used to simulate uterine spiral artery endothelium. The main components of Matrigel are laminin, collagen Ⅳ, nest protein, heparin sulfate glycoprotein, growth factors and matrix metalloproteinases. At room temperature, Matrigel was polymerized into a bioactive three‐dimensional matrix to simulate the basement membrane of cells in vivo, which could be used to study the morphology and biochemical functions of cells. In this study, we dynamically observed the spontaneous migration of HTR8/SVneo cells to HUVEC tubular structures in the gel. We further explored the specific mechanisms of action that influence this phenotype.

LncRNA‐ATB is a type of lncRNA activated by TGF‐β. Yuan et al^32^ first reported that lncRNA‐ATB was highly expressed in hepatocellular carcinoma cancer and regulated tumour invasion, colonisation and metastasis. In the subsequent years, lncRNA‐ATB was extensively studied in tumour diseases. It was found to be up‐regulated in tumours, such as osteosarcoma^33^ and lung cancer,^34^ but was down‐regulated in breast and pancreatic cancer^35^, ^36^ due to the possibility of heterogeneity of tumours or differences in detection methods. In our previous study, we reported that lncRNA‐ATB may be involved in the pathogenesis of PE,^17^ but the pathways underlying this pathogenesis have not been explored in depth. In this study, we confirmed the low expression of lncRNA‐ATB in PE although the sample size was not as large as before. Subsequently, we performed lncRNA‐ATB overexpression or knockdown experiments on HTR8/SVneo and we found that knockdown of lncRNA‐ATB resulted in a decrease in the number of HTR8/SVneo cells which replaced HUVECs. These results are consistent with results of our previous study, suggesting that lncRNA‐ATB may be involved in the process of improper spiral artery remodelling in PE that leads to the occurrence and development of PE.

Many studies have reported that lncRNA‐ATB can bind to miR‐141‐3p,^37^ miR‐195,^38^ miR‐144,^39^ and so on as competitive endogenous RNA in different diseases. Therefore, we used the database to predict some other unstudied miRNAs targets for lncRNA‐ATB. We found that miR‐651‐3p had the largest difference in expression and, therefore, focused on it for subsequent analysis. We used luciferase and qRT‐PCR assays to show that lncRNA‐ATB and miR‐651‐3p interact with each other directly. In addition, co‐transfection assays revealed that the two had opposing effects on the trophoblast‐endothelial cell network. Moreover, the RNA expression levels of the two in the placenta were negatively correlated. All the above experiments demonstrated that lncRNA‐ATB/miR‐651‐3p network may play a role of competitive endogenous RNA in the pathogenesis of PE.

Although the role of YY1 in gene regulation has been widely studied, the mechanism of YY1 action on tumour growth is distinct in different cancers.^40^ Some studies have shown that YY1 mainly acts as a tumour suppressor in pancreatic cancer,^41^, ^42^ however, most studies have shown that YY1 exists as a oncogene, such as in gastric cancer and lung cancer.^43^, ^44^ It is involved in the transcriptional activation or inhibition of many genes involved in various cellular processes, such as cell differentiation, DNA repair, autophagy, cell survival and apoptosis, and cell division.^45^ Recently, many articles have studied YY1 as a target gene for competitive endogenous RNA.^46^, ^47^, ^48^ In our study, we identified YY1 as a potential target for miR‐651‐3p and may be inversely modulated by miR‐651‐3p with methods mentioned above. Like lncRNA‐ATB, YY1 expression was down‐regulated in PE placental tissue. Trophoblast cells are thought to be similar to tumour cells in proliferation, invasion and immune tolerance.^49^ Therefore, we concluded that YY1 promoted trophoblast invasion to endothelial cells, which was also consistent with many studies on tumour diseases. Finally, we co‐transfected lncRNA‐ATB and miR‐651‐3p, and determined the protein expression of YY1 using western blot, which received the opposite regulation of lncRNA‐ATB and miR‐651‐3p. We were, therefore, able to connect the whole lncRNA‐ATB/miR‐651‐3p/ YY1 pathway.

There are still some limitations in this study. First, the sample size is not very large, because we used newly collected samples. The required sample size was calculated through the pre‐experiment, and the number of cases in the two groups are eligible. Moreover, although our experiments can simulate the spiral artery remodelling in vitro, they cannot completely replicate the microenvironment in vivo, so our experiments provide only limited insights, hence, a lot of in vivo experiments will be needed to represent this process in the future.

In conclusion, we constructed a co‐culture network of trophoblast and endothelial cells in vitro, and then verified that lncRNA‐ATB was decreased in the placenta of PE patients and could influence the process of spiral artery remodelling.

## CONFLICT OF INTEREST

The authors declare that there are no conflict of interests.

## AUTHOR CONTRIBUTIONS

**Yangxue Yin:** Conceptualization (lead); Data curation (equal); Formal analysis (equal); Investigation (equal); Methodology (lead); Software (lead); Writing‐original draft (lead); Writing‐review & editing (equal). **Jiashuo Zhang:** Data curation (equal); Resources (lead); Visualization (lead). **Hongbiao Yu:** Conceptualization (supporting); Investigation (supporting); Methodology (supporting); Resources (supporting). **Min Liu:** Data curation (supporting); Methodology (supporting); Software (supporting); Visualization (supporting). **Xuelian Zheng:** Formal analysis (supporting); Methodology (supporting); Software (supporting). **Rong Zhou:** Conceptualization (supporting); Funding acquisition (lead); Project administration (lead); Supervision (lead); Validation (lead); Writing‐review & editing (equal).

## Data Availability

The data used to support the findings of this study are available fromthe corresponding author upon request.
